# Death from Chloroform

**Published:** 1856-01

**Authors:** 


### EDITORIAL DEPARTMENT.

*Death from Chloroform.*—In our last number we noticed a case in which death occurred from the inhalation of chloroform recently in Edinburg. We regret to state that the same accident took place in this city on Saturday, Jan. 5th. We copy from the Boston Journal the following statement of the case, as prepared by Dr. Emery, who administered the chloroform.

"Between the hours of 1 and 2 o'clock on the 5th inst., I commenced to administer chloroform to Mrs. P. A. Morgan, at her request, for the purpose of removing some teeth. I commenced with a small quantity—should think from two to three drachms, on a sponge. She inhaled it without difficulty for a minute or two. Her pulse was not strong, but uniform. She then commenced to be excited, and said that I was going to extract her teeth, and she should know all about it. She said that Mrs. Paige (the lady who accompanied her) was getting the forceps to extract them with. I think about one minute had passed during this conversation and excitement. I then removed the sponge from her mouth, and in a few moments she became quiet, and satisfied that there had been no attempt made to remove her teeth. In a few moments I commenced the operation again with the same amount of chloroform. She inhaled it without difficulty about as long as she did before, and became so much excited that she got up out of the chair and insisted that I had extracted her teeth. She spit on the floor and looked to see if it was blood, and she insisted that some one was coming into the room whom she did not want to see. I sat her down in the chair again, and she then went into a spasm, closed her teeth, and breathed with difficulty. I sprinkled water on her face, and the muscles relaxed, and I asked her to get up and we would place her on the lounge. She made an effort to rise, and with my assistance stood on her feet, and then instantly sank to the floor. With the assistance of Mrs. Paige, I placed her on the lounge, and then there was a rush of blood to the brain. I sprinkled water in her face again, but she showed no signs of being conscious. Mrs. Paige went for assistance, and I immediately commenced artificial respiration by insufflation, and kept it up until Dr. Stedman came in, which was but a few minutes." To this account by Dr. E., the Journal adds—

"As was stated in our paper yesterday, the inquest was held by Dr. C. H. Stedman, and the jury returned the verdict 'that the deceased came to her death from the effects of the chloroform, and that the chloroform was a pure article, and was given at the urgent solicitation of the deceased, and with all proper care and discretion.' They further say, 'from the testimony and opinion of medical experts in this case, the jury feel compelled to caution the public against the use of chloroform, as being a dangerous anæsthetic agent.

With this recommendation we entirely agree, and we have before urged, not the necessity of caution, (for caution seems to be of no avail in these cases,) but the abandonment of chloroform and concentrated chloric ether, as anæsthetic agents, in ordinary cases; the more especially since we have the original article used for producing insensibiltty to pain, sulphuric ether, which is efficient, cheap, and above all, safe. We are not aware that any case of death has occurred from the direct effect of the inhalation of ether, and although it is possible that such an event may take place, the article is beyond all question more safe than chloroform, the number of deaths from which now amounts, we fear, to thousands.

We cannot help thinking that the amount of chloroform used in this case was very large. It appears that from "two to three drachms" were first inhaled, and that the same amount was repeated. We believe that the most approved practice in England, is to pour a few drops (twenty minims, Druitt) on a handkerchief folded into a hollow cone, or into an apparatus specially designed for the purpose, and held at the distance of a few inches from the patient's nose. This is to be repeated occasionally until anæsthesia is produced; in many cases a single drachm is sufficient.

